# CyberEye: New Eye-Tracking Interfaces for Assessment and Modulation of Cognitive Functions beyond the Brain

**DOI:** 10.3390/s21227605

**Published:** 2021-11-16

**Authors:** Michał Lech, Andrzej Czyżewski, Michał T. Kucewicz

**Affiliations:** 1 Department of Multimedia Systems, Telecommunications and Informatics, BioTechMed Center, Faculty of Electronics, Gdansk University of Technology, 80-233 Gdansk, Poland; andcz@multimed.org (A.C.); Kucewicz.Michal@mayo.edu (M.T.K.); 2 Department of Physiology and Biomedical Engineering, Mayo Clinic, Rochester, MN 55901, USA; 3 Department of Neurology, Mayo Clinic, Rochester, MN 55901, USA

**Keywords:** pupillometry, eye tracking, memory and cognition, CyberEye

## Abstract

The emergence of innovative neurotechnologies in global brain projects has accelerated research and clinical applications of BCIs beyond sensory and motor functions. Both invasive and noninvasive sensors are developed to interface with cognitive functions engaged in thinking, communication, or remembering. The detection of eye movements by a camera offers a particularly attractive external sensor for computer interfaces to monitor, assess, and control these higher brain functions without acquiring signals from the brain. Features of gaze position and pupil dilation can be effectively used to track our attention in healthy mental processes, to enable interaction in disorders of consciousness, or to even predict memory performance in various brain diseases. In this perspective article, we propose the term ‘CyberEye’ to encompass emerging cognitive applications of eye-tracking interfaces for neuroscience research, clinical practice, and the biomedical industry. As CyberEye technologies continue to develop, we expect BCIs to become less dependent on brain activities, to be less invasive, and to thus be more applicable.

## 1. Introduction to Eye-Tracking Interfaces

When we think about brain–computer interfaces (BCIs), it is hard to imagine them without processing the activity of the brain. Since the very beginning of their history, BCIs have employed various technologies to acquire, transmit, and feedback these activities invasively from inside the brain or noninvasively from its surface [1]. The information contained in these recordings has been used to sense intentions of movements, messages for communication, or even more abstract states of emotion or consciousness. One can argue that the more direct and accurate the brain recordings, the more information and the greater the possibilities for various BCI applications. Electrodes implanted directly on the brain’s cortical surface sample information-rich electrocorticogram signals or, more generally, intracranial EEG (iEEG) generated by the underlying neural networks that were utilized in a wide range of clinical applications [2,3,4,5,6,7,8,9]. BCIs based on iEEG have been applied to restore cognitive functions, including speech and communication [10,11,12,13,14]. Decoding intentions to communicate a specific character, word, or sentence is even more dependent on direct multi-channel recordings from the brain. For example, multi-channel arrays of densely packed micro-electrodes can provide accurate information to decode intentions of writing-specific characters from the alphabet and thus efficiently communicate individual words and entire sentences, including punctuation [15]. Others speculate or even report that such dense recordings of neural activities will soon be capable of reading the contents of abstract thoughts or the objects of our mental activities [10,16]. It is hard to imagine that similar tasks could be achieved with noninvasive interfaces that do not acquire activities directly from the brain.

Noninvasive interfaces based on eye movements have typically been categorized into a group of so-called human–computer interfaces (HCI). The difference is that, in contrast to BCIs, signals employed by the HCIs are not sampled directly from the brain. Tracking eye movements in the position and size of the pupil, however, contains not only information about the focus of gaze and the amount of light but also, more interestingly, about momentary cognitive states and the underlying processes in the brain. Animal studies showed that pupillometric signals could predict states of optimal cognitive performance and were correlated with momentary changes in slow and fast brain activities, and the brain’s neuromodulatory systems [17,18,19]. Hence, eye-tracking signals can indirectly sample or reflect activities in the brain, putting them at the junction between HCIs and BCIs. Human studies showed that pupillometry could track mental effort [20], memory load [21], conscious perception [22] (also possible with auditory signals [23]), or memory processing [24]. In the study by Kucewicz et al., a simple recording of pupil dilation robustly tracked encoding and recall memory items with significant differences in the remembered and forgotten trials. Given that even these raw measures of pupil size contain information about processes as complex as forming and retrieving memories of abstract concepts in our mind, eye-tracking is expected to provide an alternative or a complementary signal to current BCI applications.

## 2. Eye-Tracking BCIs for Probing Memory and Cognitive Functions

**Figure 1:**
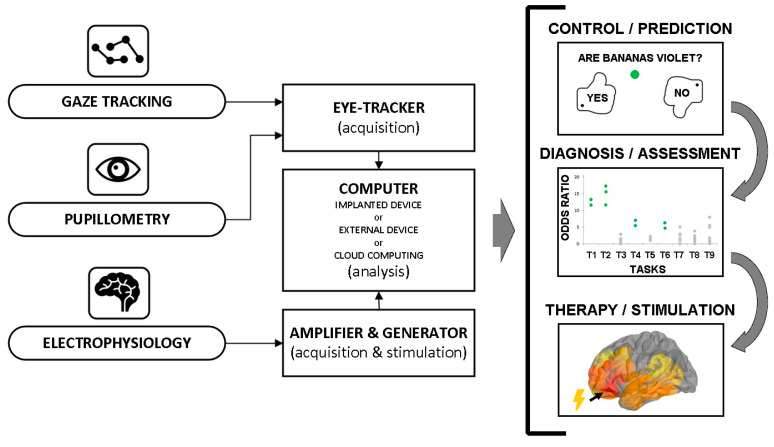
General eye-tracking BCI inputs (**left**) and outputs (**right**) complementing or providing a noninvasive alternative for the classic electrophysiological design.

In this perspective, we showcase three example applications of eye-tracking: (1) in predicting memory performance in healthy subjects, (2) in an assessment of reading comprehension in post-comatose patients, and (3) in the level of consciousness in patients with acquired brain injuries. These applications provide case studies for using the noninvasive signals from pupil size or position on a computer screen to measure cognitive processes in the brain. Hence, they fulfill the criteria for an indirect BCI mediated through the eye. It is qualitatively different from a clinical examination of the pupil or gaze responses by delivering objective measures of cognitive processing that can be used as feedback for a computer-generated response in the form of a diagnostic assessment or a therapeutic intervention such as brain stimulation. Figure 1 summarizes the general circuit design for such eye-tracking BCIs, which is common in each of the examples described below. In general, gaze tracking and pupillometric signals are sensed independently or complement electrophysiological data for computer analysis of output responses feeding back to the user or directly to the brain. An analysis of the signals and any simulations can be efficiently performed in open-access (e.g., Python) or commercially available (e.g., Matlab and MathWords Inc.) programming environments.

**Figure 2:**
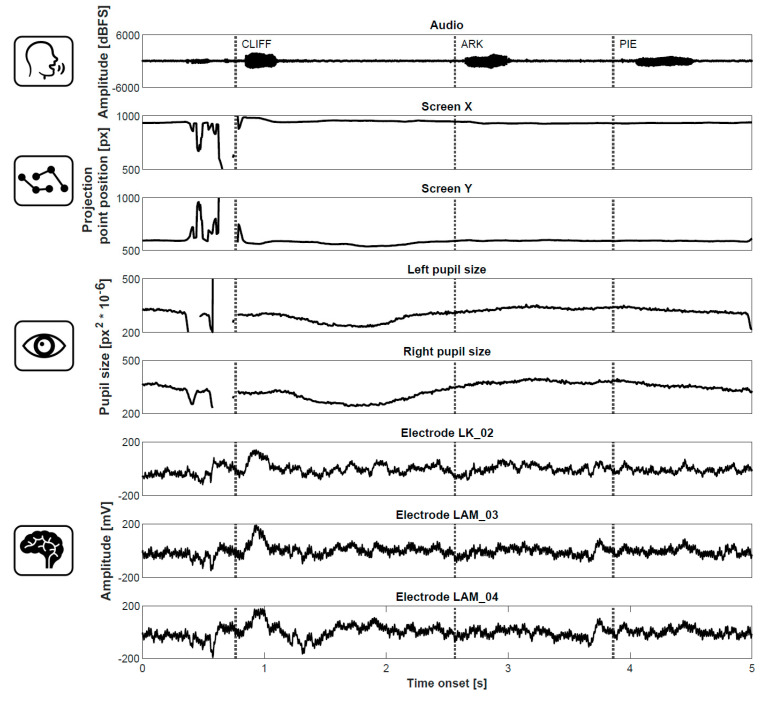
Example gaze tracking, pupillometric, and electrophysiological BCI input signals recorded in a patient with intracranially implanted electrodes during recall of three words.

In the first application, the input signal is provided by momentary fluctuations in pupil size that were shown to reflect cognitive processes engaged during memory performance [24]. A small infra-red camera placed under a computer screen where a memory task was displayed captured the *x* and *y* dimension of the pupil shape (ellipse) as the subjects remembered and then recalled previously presented word lists. The input signal was generated from high-resolution camera images sampled at rates of >100 fps and processed to detect the pupil. A subsequent computer analysis, the next stage on the diagram in Figure 1, revealed that the pupils were dilated very stereotypically and consistently with the changing cognitive demands as more words were presented on the screen. Then, memory traces for the words were held in working memory during a short distractor task. Finally, the remembered words were freely recalled out loud. The gaze positions and pupil sizes, and the intracranial, electrophysiological signals were recorded simultaneously. Figure 2 shows these signals together during recall of three words in an example recording from a patient implanted with intracranial electrodes in the brain. Each signal reveals different signatures of the cognitive processes involved in memory recall (Figure 2). The gaze position leaves the screen as the patient concentrates on and attempts to recall the first word. The pupil first constricts and then dilates as the subsequent two words are recalled, reflecting increasing mental effort used to retrieve the following two words [20,21,24]. The brain signal responses to recall of each word show a more complex pattern of oscillatory responses that requires further analysis (e.g., spectral decomposition). All in all, each signal offers a different and complementary input for a potential brain–computer interface.

As individual words were presented for memory encoding, the pupils constricted and then dilated more on the trials with subsequently remembered words compared with those that were not recalled. This memory-predictive constriction and dilation occurred at the scale of tens of milliseconds before and after word presentation, respectively. These predictive changes in the pupil input signal even before the presentation of objects to be remembered provide ideal feedback triggers for therapeutic interventions such as brain stimulation. Another potential output from this signal is an index assessing ongoing cognitive state for tasks requiring focused attention and memory that could be fed back to a user’s personal device such as a smartphone or a tablet. Most importantly, this completely noninvasive input signal can be used to predict whether a given memory successfully forms and is later retrieved, as suggested in the previous body of literature [25]. A user or a therapist can then use this information to target and direct specific therapeutic interventions—either immediately as presented or delayed at an extended timescale. Other possible applications of such pupillometric inputs on our daily lives are wide-ranging, including remembering sequences of recent events [26] or preventing attention lapses with excessive media engagement [27].

In the following two applications, the input signal was provided by coordinates of gaze position on a computer screen projected from pupil movements. The pupil is detected as in the first application, but only its geometrical position is relevant—not the size. This gaze-tracking signal was used to assess the state of consciousness in patients with acquired brain injuries [28] and reading comprehension skills in post-comatose, minimally conscious state (MCS) patients [29]. This particular solution consists of an eye tracker, two monitor displays, and one set of speakers, all connected to the regular computer with software controlling user-operated tasks. The tasks are presented to the user on one of the monitors in parallel with a control panel displayed on the other monitor for the therapist. In every task, a user selects either a word, a sentence, a digit, or an image using gaze only. While the application runs, a dot representing their gaze fixation point is projected on the screen. Thus, immediate visual feedback of motor control is provided to the user and the therapist, who can assess the saccadic movements and gaze fixations in each task. The name of the object selected is spoken by a therapist or articulated by a speech synthesizer. The selection is performed by fixing the user’s gaze on an object for 2 s. When a user indicates the object (either correctly or not), the application randomly chooses a new object. A therapist can proceed to the next set within each task using the control panel. Gaze position and timestamp information, the object name selected, and the name of the object selected by a user are registered in real-time for further analysis. This enables projecting gaze focus and controlling objects on the screen and other output feedback responses (Figure 1).

Objective assessment of cognitive processes is another example of a response that could trigger a clinical report or even treatment in the form of brain stimulation. In the presented application, when the patient is requested to select one object amongst many, the correctness of the selection can be represented on a dichotomous scale by either 1 (correct) or 0 (incorrect). This enables the responses to be statistically compared in one-tailed Fisher’s exact test with a random distribution of zeros and ones reflecting chance selection of the correct object (without awareness). The resultant values from the statistical test, i.e., the odds ratio and the corresponding *p*-value, quantify and test the level of consciousness or performance in the reading comprehension task. In this case, odds ratio values for *p* < 0.05 determine the above-chance conscious performance. The same responses in the reading comprehension skills test can also be assessed using a 5-point scale (5—ability preserved, 4—light impairment, 3—moderate impairment, 2—severe impairment, and 1—ability ceased) following the standard protocol of the speech comprehension test, as previously reported [29]. All in all, these eye-tracking measures of cognitive processes were able to detect conscious responses in patients who failed to show signs of consciousness with traditional examination methods [28]. Likewise, it was determined that patients with a minimally conscious state (MCS) preserved the partial ability of reading comprehension [29]. Such a noninvasive and objective assessment of brain functions was made possible with artificial intelligence of the eye-tracking BCIs, which complemented traditional clinical assessment tools and ultimately led to change in the clinical report and patient diagnosis. One can foresee how detecting momentary conscious responses or reading could trigger a therapist response or brain stimulation to augment and treat cognitive functions.

There are various other eye-tracking BCI applications in research and clinical studies of memory and cognition [30]. The three case studies presented in more detail in this perspective are aimed at providing a general glimpse into the present and future interfaces that probe memory, consciousness, and reading skills. Learning new information is another function that can be assessed and analyzed more automatically with gaze-tracking. Using simple measures such as the number of saccades, fixations, and blinks, it was shown that supervised and unsupervised machine-learning classification methods can provide learning profiles across different age groups [31]. Other, more abstract cognitive functions, can be probed with new eye-tracking interfaces. Artistic creativity can be expressed through a human–robot interaction, in which a robotic arm is teleoperated by projected gaze movements to draw [32]. Even an intention to select an object visually can be decoded from gaze fixation features [33]. The gaze-tracking and pupillometric features can themselves be further enhanced and complemented with other measures of head motion to provide more precise signals for robotic interfaces [34]. All in all, memory and cognitive brain functions can now be probed with a wide range of interfaces, including robotic control devices, that circumvent recording brain signals.

## 3. CyberEye—Definition and Future Perspectives

The various applications presented here are merely a few examples in a growing body of BCIs based on eye-tracking. Although signals acquired directly from the brain remain indispensable for studying the mechanisms of memory and cognition, there is now a general trend for developing more accessible technologies to assess, treat, or improve cognitive functions noninvasively. Some can even be worn with the IR camera inside special glasses [35]. Having an eye-tracking camera built in the glasses opens up opportunities for individualized systems, e.g., with corrections of convex and concave lenses for specific visual impairments. Still, compared with traditional BCIs, these technologies are limited in terms of the amount of information that can be mined from eye-tracking signals and the possibility to modulate cognitive processing in the brain. For instance, a BCI for communication-based on an intracranially implanted grid of electrodes decoded cortical motor commands for handwriting specific letters and characters to typing sentences at speeds greater than those achieved by keyboard typing with gaze-tracking interfaces [15]. The rates were comparable to text messaging on smartphones. Whether specific letters or words could be as quickly decoded from patterns of macro- and micro-scale eye movements (saccades and microsaccades) remains yet to be determined. In general, direct recording and modulation of brain activities provide greater opportunities than eye-tracking interfaces alone. Combining the two may prove to be a powerful augmentation to classic BCI designs for treating memory and cognitive functions (Figure 1 and Figure 2). It could, for instance, improve the classification of cognitive states to enhance memory performance [36,37,38]. A noninvasive prediction of cognitive states from eye-tracking can thus be utilized in emerging brain stimulation technologies that target memory and cognition.

In addition to describing a new trend for more accessible noninvasive interfaces, we propose a new term, ‘CyberEye’, to define the various BCIs based on eye-tracking that target cognitive functions. This class of BCIs is characterized by providing a window or ‘an eye’ to the brain’s internal processes and the mind [39]. The ‘CyberEye’ BCIs effectively exchange information between the ‘internal’ brain and the ‘external’ computer processes. In other words, the internal processes become available to be externalized and distributed to the virtual reality of local devices or remote cloud computations. Distributed processing of neural signals has already been applied in the management of seizures in epilepsy [40,41,42] and could analogously be extended to cognitive functions. CyberEye technologies enable the distribution of the cognitive processing reflected in pupillometric signals, which are used without the need to record neural activities from the brain. They could be conceptualized as a noninvasive computer sensor of information about the internal mental states that are made available for distributed external processing. One could think of a whole range of implementations in addition to the ones showcased here for machine learning and artificial intelligence tools to classify states of consciousness, attention, memory, or specific mental contents. Allegorically, we propose that CyberEye is the interface where human and artificial intelligence meet beyond the brain.

## Data Availability

No new data were created or analyzed in this study. Data sharing is not applicable to this article.
